# Limits to Forecasting Precision for Outbreaks of Directly Transmitted Diseases

**DOI:** 10.1371/journal.pmed.0030003

**Published:** 2005-11-22

**Authors:** John M Drake

**Affiliations:** **1**National Center for Ecological Analysis and Synthesis, Santa Barbara, California, United States of America; Harvard Medical SchoolUnited States of America

## Abstract

**Background:**

Early warning systems for outbreaks of infectious diseases are an important application of the ecological theory of epidemics. A key variable predicted by early warning systems is the final outbreak size. However, for directly transmitted diseases, the stochastic contact process by which outbreaks develop entails fundamental limits to the precision with which the final size can be predicted.

**Methods and Findings:**

I studied how the expected final outbreak size and the coefficient of variation in the final size of outbreaks scale with control effectiveness and the rate of infectious contacts in the simple stochastic epidemic. As examples, I parameterized this model with data on observed ranges for the basic reproductive ratio (*R*_0_) of nine directly transmitted diseases. I also present results from a new model, the simple stochastic epidemic with delayed-onset intervention, in which an initially supercritical outbreak (*R*_0_ > 1) is brought under control after a delay.

**Conclusion:**

The coefficient of variation of final outbreak size in the subcritical case (*R*_0_ < 1) will be greater than one for any outbreak in which the removal rate is less than approximately 2.41 times the rate of infectious contacts, implying that for many transmissible diseases precise forecasts of the final outbreak size will be unattainable. In the delayed-onset model, the coefficient of variation (CV) was generally large (CV > 1) and increased with the delay between the start of the epidemic and intervention, and with the average outbreak size. These results suggest that early warning systems for infectious diseases should not focus exclusively on predicting outbreak size but should consider other characteristics of outbreaks such as the timing of disease emergence.

## Abstract 2

Using a mathematical model, John Drake shows that early warning systems for infectious diseases should not focus exclusively on predicting outbreak size but should consider other characteristics of outbreaks such as the timing of disease emergence.

## Introduction

The epidemiological responsibility to forecast disease outbreaks is an onerous one. Because of the devastating consequences and high costs of disease, predicting outbreaks is a chief goal for public-health planning and emergency preparedness. Thus, quantitative forecasting and development of early warning systems (EWSs) for disease outbreak is a high priority for research and development [1]. According to the World Health Organization, the primary goals of EWSs are to predict the timing of the outbreak and the magnitude of the outbreak [1]. Intuition suggests that for directly transmissible diseases, the magnitude of the outbreak will be extremely difficult to predict because of the stochastic process of infectious contacts [2,3]. This idea is consistent with the recent finding of Sultan et al. [4] that although the timing of annual meningitis outbreaks in West Africa was highly predictable, the final outbreak size varied greatly from year to year. Here I study fundamental limits to forecast precision for (eventually) controlled outbreaks, first theoretically, then using nine well-studied infectious diseases as examples. Finally, I consider a new model that more realistically represents actual outbreaks of emerging infections.

The reason that final outbreak size is generally not predictable is that the eventual dynamics of the outbreak are highly sensitive to the seemingly random sequence of infectious contacts and removal of infectious individuals in the early, typically unobserved stages of the outbreak [3]. Clearly, the final size of an outbreak depends on numerous aspects of the social structure of the population, the environment, and disease- or strain-specific characteristics. Among the more important factors are seasonal climate fluctuations, transmissibility and virulence of the pathogen, population dynamics and structure of the host population, physiological and immunological status of potential hosts, and the social networks of contacts between infectious and susceptible individuals [5–8]. Accordingly, the deterministic approach to epidemic modeling regards the spread of infectious diseases as completely determined by the average effects of these factors on the basic reproductive ratio (*R*_0_) together with initial conditions. Deterministic models of epidemics have provided insight into such important topics as the design of vaccination campaigns and the effect of age structure on epidemic dynamics [5]. From the perspective of EWSs, the timing and average severity of outbreaks also might be modeled quite accurately with deterministic models. However, for emerging diseases or for diseases prone to sudden outbreak, numerical predictions of final outbreak size derived from deterministic models will often deviate substantially from the observed outbreak size [3,9].

In contrast, the stochastic theory of epidemics represents the population as a statistical ensemble with constant or regular average properties but probabilistic changes in disease status for individuals. As a result, properties of the ensemble, such as the final epidemic size, are probabilistic as well [10–14]. Thus, stochastic models quantify the likelihood of outbreaks that deviate from the expected final size [9,15]. Such information about the variation in final outbreak size—its predictability—is crucial if disease forecasting is to be relied upon for planning interventions.

The stochastic theory of epidemics can therefore be used to understand the theoretical limits to forecasting precision for disease outbreaks, including EWSs or forecasts based on the developing epidemic curve as case reports accumulate. I studied how precision in the forecasted final outbreak size for transmissible diseases depends on two dynamical features of outbreaks: the contact rate (β) and the rate of removal (γ) in the simple stochastic epidemic. Next, I developed models of forecast precision for nine outbreak-prone diseases (chicken pox, diphtheria, measles, mumps, poliomyelitis, rubella, scarlet fever, smallpox, and whooping cough) and used removal rate as a control parameter to relate intervention effectiveness to final outbreak size and forecast precision. Finally, I developed a new model to understand how delays in implementing interventions affect final outbreak size and forecast prevision.

## Methods

### Model

The simplest realistic model for outbreaks with a small number of initially infectious individuals is the simple stochastic epidemic with contact rate β and removal rate γ, which do not change appreciably over the time scale of the outbreak [15,16]. This model is a good approximation if the outbreak meets the following criteria, which are reasonable for modern outbreaks that are rapidly controlled. First, we assume that infectious contacts and removal of infectious individuals are approximately independent in time so that the outbreak is Markovian (compare [17–19]). Second, the rate at which infectious individuals are removed from the population exceeds the rate at which infectious contacts occur (β < γ). Finally, the population is sufficiently large that the number of individuals ultimately infected is not more than a negligible fraction of the susceptible population (i.e., per capita transmission rates are approximately independent of the density of infected individuals). Then, the outbreak is a homogeneous birth–death process (the simple stochastic epidemic) with mean *(M)* and variance *(V)* of the final outbreak size given by [10]:



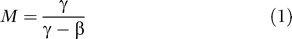



and



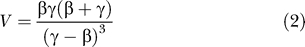



Properties of the final size distribution for other classes of epidemics can be found in [11–14,17,20].

The solution given by equations 1 and 2 is for an outbreak in which either (1) epidemiological parameters are naturally such that always *R*_0_ < 1, or (2) public health policy is applied consistently so that intervention is constant and under policy conditions *R*_0_ < 1. For many emerging diseases this is not the case. Rather, initially *R*_0_ > 1, but through intervention that was established some measurable time after the outbreak started, the reproductive ratio is reduced below the epidemic threshold (e.g., severe acute respiratory syndrome [SARS]). This case is considerably more complicated and, to my knowledge, no simple formulas have been obtained for the mean and variance of the final outbreak size. However, it is reasonably straightforward to solve the equations computationally, and a range of conditions can be studied. Below, I consider a case that is more applicable to forecasting emerging diseases, the simple stochastic epidemic with delayed-onset intervention in which there is a constant rate of infectious contacts (β) and a removal rate (γ) that depends on the time since the outbreak began. Specifically, at the start of the outbreak the removal rate is some value less than the rate of infectious contacts and remains constant until some intervention is applied a time t*−*t*_0_ later, after which the removal rate is some constant value greater than β, i.e., γ(*t*) = γ_1_ *I*(*t*≤*t* ^*^) + γ_2_ *I*(*t*>*t* ^*^) , where *I* is an indicator function equal to one if its argument is true and zero otherwise. Then, we can study the size of the outbreak as a function of the control parameter t*, the time at which intervention is initiated.

### Analysis

A measure of precision should quantify the relative magnitude of deviations from an expected value. The coefficient of variation 
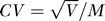
 is a measure of forecast precision that can be interpreted as relative dispersion independent of the magnitude of the data [21]. I used the theoretical CV for final outbreak size obtained from equations 1 and 2, which depends only on the ratio γ/β = *R*_0_^−1^ and not on the individual parameter values, to study how forecast precision depends on outbreak characteristics and to estimate forecast precision for nine infectious diseases under different levels of control, represented by increasing γ (see Figure S1). This measure assumes β and γ are known exactly. For individual outbreaks, in which β and γ are not precisely known and the model is only an approximation to the structure of the contact process, violations of modeling assumptions such as the Markov assumption and the lack of an explicit incubation period further erode forecast reliability. Thus this measure represents a theoretical upper bound on forecast precision that will not be attainable in practice.

Although every outbreak will be different as a result of evolution of the etiological agent, changes in social behavior, timing, and the ecological and geographical context in which the outbreak starts, many epidemic parameters (most famously *R*_0_), are reasonably conserved across outbreaks of the same disease. Here, I treat the removal rate γ as a control parameter because it is crucially related to interventions, and estimate β, which is assumed to depend on uncontrollable aspects of the outbreak. The variable β, which is the individual rate of infectious contacts [22], is related to the transmission rate (β_0_) by the equation β = β_0_ *N,* where *N* is total population size or density in the standard theory (e.g., [5]). This quantity is related to the basic reproductive ratio *R*_0_ by the equation:



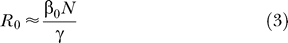



Where removal results from recovery of the diseased individual, we can estimate γ from the duration of the incubation (τ_1_), latent (τ_2_), and infectious (τ_3_) periods with the equation γ = (τ_1_+τ_2_+τ_3_)^−1^ . Estimates of *R*_0_ have been obtained for numerous directly transmitted diseases [5]. Assuming these estimates are based on the natural course of the disease (i.e., without direct intervention), we can rearrange this equation and substitute for γ to obtain an estimate of β:



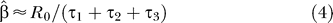



Given that reported values for these variables vary somewhat, we put an upper bound on β by choosing the highest reported value of *R*_0_ and the lowest reported values for the different τs, whereas a lower bound is obtained from the lowest reported value of *R*_0_ and the highest reported values for the different τs. As a central estimate, I used the center of the reported interval for each variable. Estimates of the ranges of these quantities for several directly transmitted diseases were compiled by Anderson and May ([5], Tables 3.1 and 4.1). Using these values, I used equation 4 to estimate plausible ranges of β for nine directly transmitted diseases (Table 1).

**Table 1:**
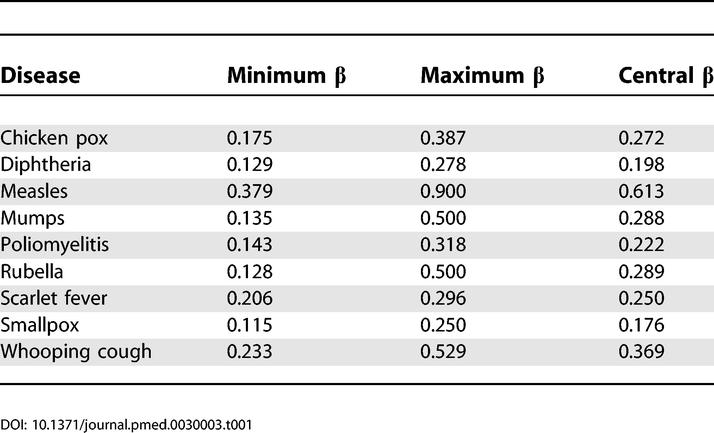
Estimates of the Range of β for Nine Directly Transmitted Diseases

I also considered the delayed-onset intervention model wherein initially β > γ (the supercritical case in which epidemic occurs with high probability), but after a time t*−*t*_0_ intervention increases the removal rate γ so β < γ (the subcritical case in which the outbreak is brought under control). This model is a more realistic representation of many emerging outbreaks (e.g., SARS, Foot-and-Mouth disease, and Marburg virus). The solution to the simple stochastic epidemic with delayed-onset intervention can be obtained using generating functions for the probability distribution of the size of the outbreak [10]. The variance of the final outbreak size is in terms of a multiple integral, which was evaluated numerically (see Text S1). As an example, I studied two situations with contrasting initial values for *R*_0_. First, I studied the situation with β = 0.5 and γ_1_ = 0.25 (*R*_0_ = 2). Second, I studied the situation with β = 0.5 and γ_1_ = 0.45 (*R*_0_ ≍ 1.1). In both cases, γ_2_ (the removal rate after intervention) was one, so that post-intervention reproductive ratio was 0.5.

## Results

**Figure 1:**
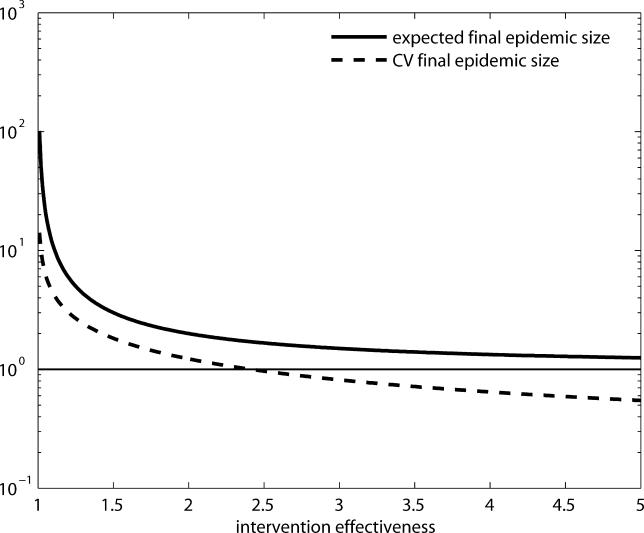
Expected Final Outbreak Size and CV in the Final Outbreak Size as a Function of Intervention Effectiveness The expected final outbreak size (solid line) and CV in the final outbreak size (dashed line) are shown as a function of intervention effectiveness (the ratio of the removal rate and contact rate γ/β) for the simple stochastic epidemic. The light horizontal line designates the benchmark where CV = 1.

**Figure 2:**
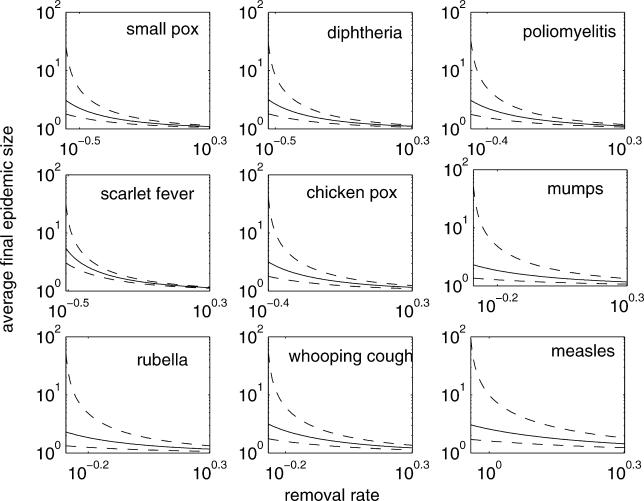
Expected Final Outbreak Size for Nine Directly Transmitted Diseases as a Function of the Removal Rate The expected final outbreak size (*y*-axis) for nine directly transmitted diseases is represented as a function of the removal rate (*x*-axis). Estimates are bounded by minimum and maximum estimates (dashed lines) of the contact rate β based on published estimates of *R*_0_.

**Figure 3:**
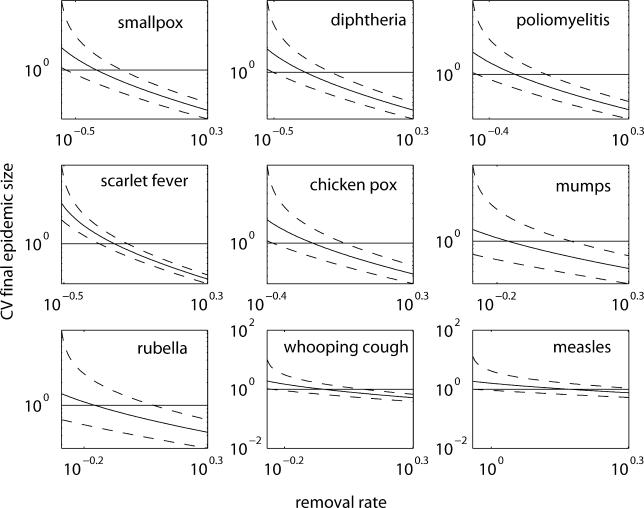
CV in Final Outbreak Size as a Measure of Forecast Precision for Outbreaks of Nine Directly Transmitted Diseases as a Function of Removal Rate The CV in final outbreak size (*y*-axis) is a measure of forecast precision, shown here for outbreaks of nine directly transmitted diseases as a function of removal rate (*x*-axis). Estimates are bounded by minimum and maximum estimates (dashed lines) of the infectious contact rate β based on estimates of *R*_0_. The horizontal line indicates CV = 1.

The ratio γ/β, the rate of removal compared with the rate of infection, represents the relative effectiveness of interventions. In the simple stochastic epidemic, the relative effectiveness of intervention is always greater than one because we assume that the outbreak is eventually controlled, i.e., the assumption β < γ above. Figure 1 confirms the intuition that final outbreak size declines as the relative effectiveness of intervention is increased. The CV in the final outbreak size, our measure of the imprecision with which the final outbreak size is forecasted, also declines with control effectiveness. As a benchmark, a forecast might be deemed reliable (in principle) where the CV is less than one, which occurs for 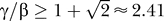 . Figures 2 and 3 show plots of the final outbreak size and the CV over the interval of estimated βs for each of nine directly transmitted diseases. It is important to underscore that the intervals in Figures 2 and 3 represent uncertainty about the value of the parameter β, not variation from stochastic fluctuations. Further understanding of these diseases might allow us to reduce this source of uncertainty by obtaining more precise estimates. In contrast, the CV in Figure 3 represents the range of final outbreak sizes that can result from the stochastic infection process for a fixed set of parameters. In principle, no amount of detailed information about transmission or other ensemble epidemic parameters can reduce this uncertainty.

**Figure 4:**
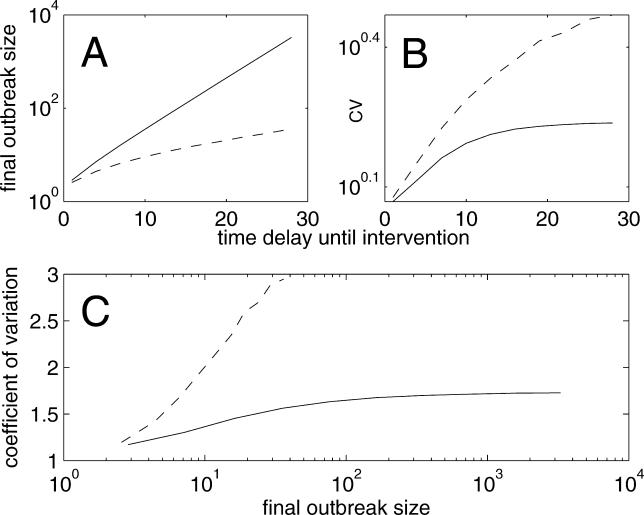
Effect of Time Delay until Intervention on Outbreak Size Effect of time delay until intervention on outbreak size is contrasted for outbreaks with *R*_0_ = 2 (solid lines) and *R*_0_ ≍ 1.1 (dashed lines). (A) Average outbreak size (*y*-axis) increases with the number of days until intervention (*x*-axis). (B) CV in outbreak size (*y*-axis) increases with the number of days until intervention (*x*-axis). (C) CV in outbreak size (*y*-axis) increases at a declining rate (i.e., levels off) as the average outbreak size increases (*x*-axis). Note that the CV in final outbreak size increases faster in the outbreak with lower *R*_0_.

Numerical analysis of the delayed-onset intervention model showed that (1) the average outbreak size increased with the delay between the start of the outbreak and the start of intervention (Figure 4A), and (2) the CV (in our examples) was everywhere greater than one and increased with the time delay between the start of the outbreak and intervention, but at a declining rate (Figure 4B). The first result is straightforward: The delay between initial infection and intervention increases the total number of secondary (tertiary, etc.) infections that are increasing as a multiplicative process. The explanation of the second result is that the CV in outbreak size scales as the square root of the variance in outbreak size and as the inverse of the average outbreak size. As the average outbreak gets larger the CV increases but at a declining rate (Figure 4C). This effect is mediated by the reproductive ratio of the outbreak, so that the outbreak with the lower *R*_0_ had a lower average outbreak size (Figure 4A), but larger CV (Figure 4B and 4C). Thus, in the sense that the CV measures the predictability of the outbreak, we found that subcritical and controlled outbreaks (*R*_0_ < 1 and *R*_0_ close to 1, respectively) were less predictable (have lower CV) than supercritical (*R*_0_ >> 1) outbreaks of comparable size.

## Discussion

Using theoretical models, I found that unless controls are extremely effective, limits to forecast precision result in highly uncertain estimates of final outbreak size. Specifically, for the simple stochastic epidemic (subcritical case), unless the removal rate is greater than approximately 2.41 times the effective contact rate, the CV of final outbreak size will be greater than one. Imprecision in the delayed-onset intervention model was typically even greater.

Reliable forecasts of outbreaks based on initial cases and/or EWSs could potentially save many lives by increasing preparedness for outbreaks when and where they are most likely or most severe. According to the World Health Organization, forecasts will be most useful when they accurately predict the final size of the outbreak [1]. However, the findings reported here suggest that precise predictions may be unattainable because of high variance in the final outbreak size of directly transmissible diseases, even under the (unreasonable) assumption of perfect information about macroscopic epidemic parameters.

This result does not apply to diseases that are not directly transmitted (e.g., vector-borne illnesses) or to diseases in which parameters change as the outbreak progresses (e.g., SARS [23]). Parameters might change for at least two reasons. First, for emerging infections, about which little is known at the start of the outbreak, increasing ability to diagnose and treat infected patients and the dissemination of information to the public will result in increasing the removal rate. Thus, for example, in the 2003 SARS outbreak, the average lag between onset of symptoms and hospital isolation was initially around 6 d but declined to around 2 d by the fourth wk of the outbreak [23,24]. Second, in outbreaks that ultimately infect a large portion of the population, the rate of infectious contacts will decline as the number of cases increases, diluting the susceptible population. These examples represent important violations of modeling assumptions adopted here and are represented by the inhomogeneous [10,22] and general [15,16] stochastic epidemics respectively. Forecasting precision for these situations is an important topic for research.

Generally, these violations of the simple stochastic epidemic must be considered on a case-by-case basis. We studied one realistic example (the simple stochastic epidemic with delayed-onset intervention) in which an initially supercritical outbreak (*R*_0_ > 1) is controlled by public health measures that increase the rate at which infectious individuals are removed from the population to a level ensuring the outbreak will eventually die out. This is a reasonably realistic model for dynamics of emerging infections with a short incubation period. For two representative examples, we found that the average outbreak size scaled approximately exponentially with the delay between the start of the outbreak and the implementation of intervention (note the log scale of the *y*-axis in Figure 4A), underscoring the importance of rapid intervention. Intuitively, when *R*_0_ was high the average outbreak size increased faster than when *R*_0_ was low. We also found that the CV in the final outbreak size increased with the lag between initial infection and control, but was smaller in the case with high *R*_0_ than in the case with low *R*_0_. Indeed, for the delayed-onset case with relatively high *R*_0_ (*R*_0_ = 2) the CV seemed to level off at a delay of around 15–20 d, although this was not shown in the case with lower *R*_0_ (Figure 4B and 4C), probably because a longer delay would be required to reach such an asymptote.

In conclusion, the fundamental limit to forecasting precision obtained here represents only variation that results from the stochastic contact process and not from uncertainty about the underlying model or parameter values (compare [3]). These sources of uncertainty will further diminish precision. Further, these results underscore that rapidly implementing control measures has value not only for decreasing the final size of the outbreak, which is the primary goal, but also for decreasing variation in the final size of the outbreak, which is information that can be used to tailor control measures and reduce potential losses. Although these limits to forecast precision should lead to interpreting predictions cautiously—whether derived from statistical analysis, epidemic modeling, computer simulation, or expert opinion—they should not hinder the development of greater and more reliable systems for forecasting outbreaks of infectious disease because there are many features of outbreaks that might be reliably predicted.

## Supporting Information

Figure S1 CV of Final Outbreak Size as a Function of *R*_0_
I was unable to obtain a simple relation for the coefficient of variation (CV) in the outbreak size of the subcritical simple stochastic epidemic in terms of the basic reproductive ratio *R*_0_. Numerical results confirm that the CV of final outbreak size depends only on the ratio β and γ (i.e., on *R*_0_). This plot represents the information in Figure 1 as a function of *R*_0_. The value  is the value at which CV equals exactly one. In this sense, outbreaks with *R*_0_ ≤ *R*_0_* are predictable while outbreaks with *R*_0_ > *R*_0_* are unpredictable.
(18 KB PDF).

Text S1 Numerical Methods to Obtain Variance in Outbreak Size in the Delayed-Onset Intervention Model (102 KB PDF).

**Patient Summary**

**Background**

Early warning systems that are used to look for outbreaks of infectious diseases are important in public-health planning. One of the most important things that such early warning systems try to predict is the final size of the outbreak. However, for diseases transmitted directly from person to person (rather than via a mosquito, for example), the precision with which the final size can be predicted is often very low.

**Why Was This Study Done?**

This researcher wanted to study how predictable the final outbreak size of an epidemic is if the effectiveness of control measures and the average number of infectious contacts are known.

**What Did the Researcher Do and Find?**

He developed a mathematical model that took into account the variation in the infectiousness of nine well-studied infectious diseases. He found that for any outbreak that increases slowly, precise forecasts of the final outbreak size will be impossible. This result was especially true for epidemics in which there was a substantial delay in intervention after infection occurred, and the precision of the forecast got worse as the delay between the start of the epidemic and intervention increased, and with the average outbreak size.

**What Do These Findings Mean?**

These results suggest that early warning systems for infectious diseases should not focus just on trying to predict outbreak size because this estimate may be inaccurate, but rather they should instead try to predict other characteristics of outbreaks. These results will be of use to people trying to plan for infectious disease outbreaks, but will not affect how patients are managed individually.

**Where Can I Get More Information Online?**

Based in the United States, the Centers for Disease Control and Prevention (CDC) has a Web site that gives background on how the CDC investigates disease outbreaks, along with details of individual diseases:

http://www.cdc.gov

The World Health Organization has interesting information on early warning systems:

http://www.who.int/csr/alertresponse/en/

In the United Kingdom, the Health Protection Agency has a similar function and gives details on investigations of infectious diseases:

http://www.hpa.org.uk/infections/

## Abbreviations

CV: coefficient of variation
EWS: early warning system
SARS: severe acute respiratory syndrome
