# Supplementary material for: Limits to Forecasting Precision for Outbreaks of Directly Transmitted Diseases
Source: PLoS Med. 2005 Nov 22;3(1):e3. doi: 10.1371/journal.pmed.0030003 (PMC1288026; doi:10.1371/journal.pmed.0030003)
Supplement: Supplementary file 1 [file pmed.0030003.sg001.pdf]

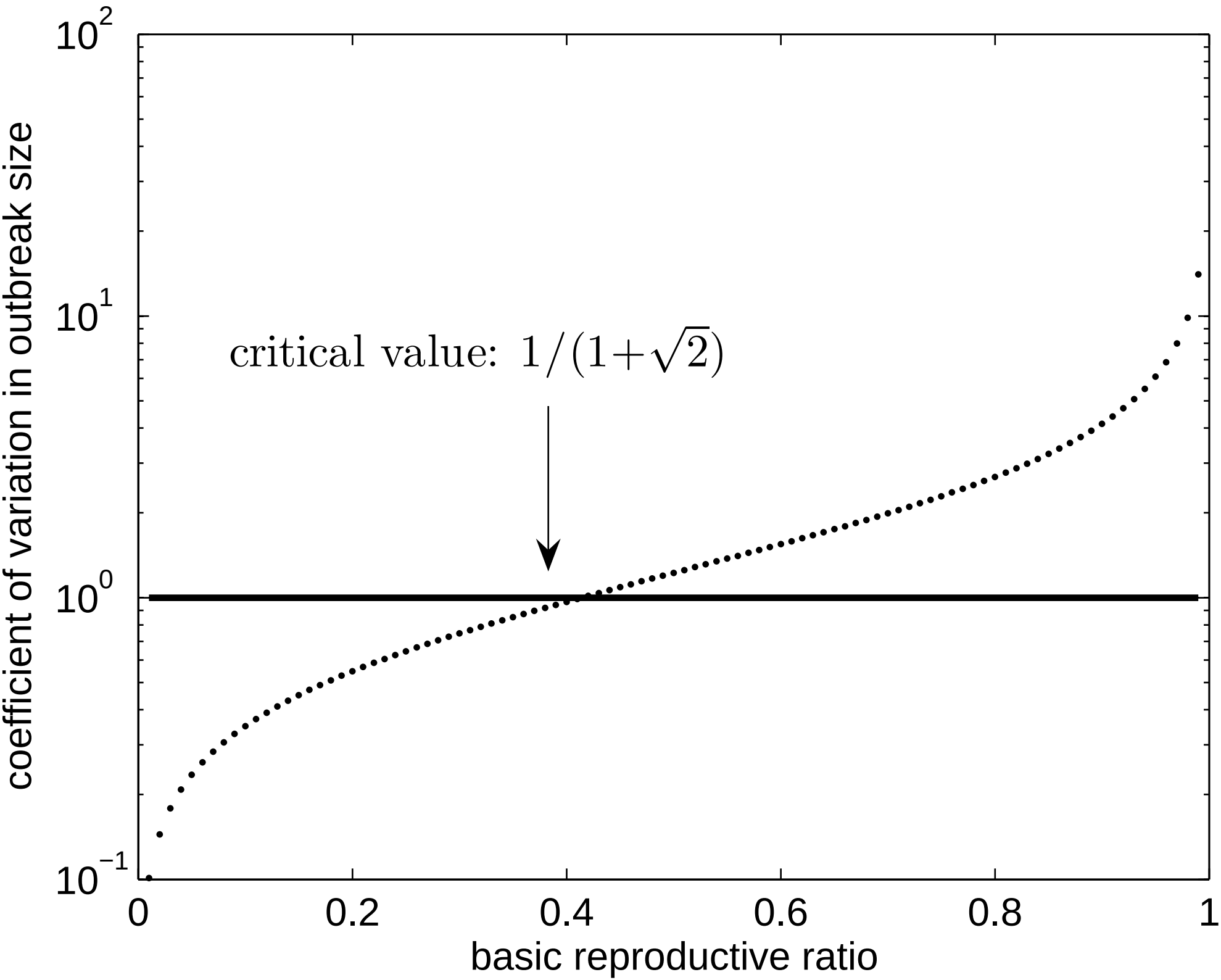

critical value: $1/(1+\sqrt{2})$
coefficient of variation in outbreak size
basic reproductive ratio
10^{-1}
10^{0}
10^{1}
10^{2}
0
0.2
0.4
0.6
0.8
1
