# Supplementary material for: Limits to Forecasting Precision for Outbreaks of Directly Transmitted Diseases
Source: PLoS Med. 2005 Nov 22;3(1):e3. doi: 10.1371/journal.pmed.0030003 (PMC1288026; doi:10.1371/journal.pmed.0030003)
Supplement: Supplementary file 2 [file pmed.0030003.st001.pdf]

Supplementary Information Text S1.

Making only small changes to the notation of Kendall (1948; ref. [10] in the main text), the variance in the size of the outbreak at time t is given by the formula

(S1) $$V = \beta_0 \int_0^t \{\bar{n}_\tau + 2Cov(n_\tau, X_\pi)\} d\tau ,$$

where $\bar{n}_t$ is the average number of infected individuals at time t and Cov is the covariance between the number of infectious individuals at t and the total size of the outbreak at t. Of course, for outbreaks that are eventually controlled, the number of infected individuals and covariance will both be brought to zero and the total outbreak size will converge on a finite quantity. Thus, practically speaking, eqn S1 need not be integrated to infinity but just to a large time t_{large} longer that the duration of the typical outbreak. Now, working "backward", S1 in turn depends on the following quantities: the covariance

(S2) $$Cov = \bar{n}_t \beta_0 \int_0^t \{1 + V(n_\tau)/\bar{n}_\tau\} d\tau$$

depending on the variance in the number of infectious individuals at t,

(S3) $$V(n_t) = e^{-2\rho(t)} \int_0^t e^{\rho(\tau)} \{\beta_0 + \gamma(\tau)\} d\tau ,$$

where

(S4) $$\rho(t) = \int_0^t \{\gamma(\tau) - \beta_0\} d\tau .$$

The average total outbreak size is given by

(S5) $$M = 1 + \beta_0 \int_0^\infty e^{-\rho(\tau)} d\tau .$$

As above, M converges at a time $t<\infty$ when infectious individuals are removed from the population.

For the model with removals given by $\gamma(t)=\gamma_1 I(t \le t^*)+\gamma_2 I(t > t^*)$, the solution to S1 was computed in MATLAB 7.0.1 R14 (MathWorks, Inc., Natick, Massachusetts) using recursive adaptive Simpson quadrature (function `quad`) for the integral in eqn S3 and the trapezoidal rule (function `trapz`) with step size $\tau=0.1$ for the integrals in eqns S1 and S2.
